# Surgical Ventricular Restoration: An Operation To Reverse Remodeling - The Basic Science (Part I)

**DOI:** 10.2174/157340309789317878

**Published:** 2009-11

**Authors:** Ganesh Shanmugam, Imtiaz S Ali

**Affiliations:** Department of Surgery, Division of Cardiac Surgery, Dalhousie University, QEII Health Sciences Centre, Halifax Infirmary, 1796 Summer Street, Halifax, Nova Scotia B3H 3A7, Canada

**Keywords:** Surgical ventricular restoration, remodeling, heart failure.

## Abstract

Congestive heart failure as a consequence of ischemic heart disease is an increasing medical problem. Notwithstanding the huge advances in the medical and conventional surgical management of heart failure, eventual outcomes remain suboptimal. This 2 part article outlines the magnitude of the problem, the limitations of conventional therapies as they exist, and the use of newer procedures that directly address the restoration of ventricular pump function.

The first part of the article deals with the pathology of different facets of the remodeling process, and the unique anatomy, geometry and flow dynamics as they pertain to ventricular function in the normal as well as the failing heart. It then details the limitations of conventional therapy, thereby laying the basis for the need and evolution of newer surgical procedures and ends with the selection of patients for ventricular restoration procedures and the pitfalls in the choice of patients for such newer techniques.

## 1. INTRODUCTION

Heart failure [HF] affects approximately 5 million Americans [1] and claims 700,000 lives a year, notwithstanding the wealth of resources expended in addressing this problem. HF may be systolic or diastolic. The commonest cause of systolic HF is ischemic heart disease [IHD]. Coronary artery disease [CAD], myocardial infarction [MI], and subsequent ischemic cardiomyopathy is the etiology of congestive heart failure [CHF] in approximately two thirds of existing cases [2]. IHD may result in left ventricular [LV] systolic dysfunction due to myocardial stunning, hibernation or infarction. In contrast to stunning and hibernation, LV systolic dysfunction after myocardial infarction is characterized at least partly by nonviable myocardium which may not improve with revascularization.

## 2. REMODELING

Acute MI causes sequential myocyte necrosis from endocardium to epicardium. [3]. Emergent revascularization by thrombolysis, percutaneous transluminal coronary angioplasty [PTCA] or coronary artery bypass grafting [CABG], salvages epicardial muscle but does not prevent necrosis of the inner and mid myocardial layers if reflow is delayed. Consequently, transmural infarction and dyskinesia are uncommon. Instead the myocardium retains its thickness and becomes akinetic. Unfortunately infarct zone change from dyskinesia to akinesia does not always improve regional contractility.

### 2A. Mechanical Remodeling

The geometric adaptations following acute MI constitute LV remodeling and are characterized by increases in LV end-diastolic and systolic volumes, wall thinning, increased sphericity and progressive worsening of cardiac function resulting in CHF [4,5]. Ventricular remodelling involves structural, cellular, extracellular, molecular, biochemical and metabolic mechanisms. Variables that determine LV remodeling include infarct size, transmurality, infarct location, loading conditions, previous scarring and revascularization [5]. Post infarct remodeling includes: (1) non-contractile and potentially expanding scar in the infarcted zone, (2) the volume load induced by such expansion and (3) the pressure load induced by the increased volume load. Additional ischemic insults contribute to remodeling. Thus, a mixed pressure and especially volume load exists [6, 7] with remodelling, with a consequent fall in EF in proportion to infarct size [8]. LV dilatation augments stroke volume by the Starling mechanism to maintain cardiac output. Early ventricular dilation is an adaptive mechanism, is beneficial and promotes survival but has deleterious long-term hemodynamic consequences.

Dilatation increases wall tension by Laplace's law. LV wall stress/tension is proportional to the radius and pressure within the LV chamber and inversely proportional to LV wall thickness. Increasing LV volume translates to increasing myocyte stress, which impedes effective contraction. The augmented wall stress results in increased oxygen consumption, decreased subendocardial blood flow and reduced systolic shortening. LV function may deteriorate toward cardiogenic shock in the early phase or may eventually lead to severe HF. Increased sphericity leads to MR. LV dilatation and MR are linked in a vicious cycle whereby MR causes dilation that in turn changes ventricular shape, thereby promoting MR. The sphere becomes a unifying geometric concept that arises from ischemia, valve insufficiency or myocyte disease.

LV dilatation in ischemic cardiomyopathy falls into three categories:

- Dyskinetic aneurysms from extensive scar in the absence of reperfusion - The commonest cause of an LV aneurysm is acute occlusion of the LAD, with aneurysm formation in the anterior wall and septum. Development of a LVA leads to heart failure, VT and thromboembolic events.
- Akinetic extensively scarred regions following early reperfusion with epicardial salvage and
- Akinetic areas occurring late due to remote muscle dilation after early aneurysm formation - These patients have significantly dilated poorly contracting left ventricles but without massive scars.

### 2B. The Biomechanical Model of HF

Medical therapy acting against neurohumoral activation slows progression but fails to arrest remodeling. When LV volume has increased beyond a certain extent and the geometry is markedly abnormal, HF progresses independently of neurohumoral activation, according to the biomechanical model of HF expressed by Mann and Bristow [9].

At some point in the natural history of the disease, a transition occurs that takes the disease beyond the limits of conventional medical or surgical therapy. The concept of a biomechanical model of HF introduces the need for procedures such as SVR that reduce LV volumes and restore geometry.

### 2C. Electrical Remodeling

Ischemic cardiomyopathy [ICMPY] is characterized by inter and intraventricular conduction delay. Interventricular conduction delay produces RV-LV dyssynchrony. Electrical resynchronization improves LV performance by avoiding right ventricular septal displacement and limiting presystolic MR [10-12].

LV dyssynchrony may involve different components of the LV mass to produce intraventricular dyssynchrony [13] Ischemic scar causes tissue inhomogeneity in dyskinetic and akinetic muscle, where non-uniform contraction, relaxation and filling may develop, thereby contributing to deterioration of global systolic and diastolic function. 

### 2D. Mechanical Performance in Ischemic Cardiomyopathy

Systolic lengthening occurs during the isovolumic phase and is caused by stretching of the ischemic/scarred segments by the adjacent normally contracting segments. Energy is expended as normal segments contract to produce pressure. The ischemic/scarred segment is unable to generate sufficient tension and thus passively bulges. The consequence is dissipation and wasting of energy produced by normal segments, which stretch the ischemic/scarred tissue and therefore does not contribute to ejection [14].

Pressure volume loops show abnormalities in morphology, size and orientation. The most common abnormalities are early shortening and early relaxation, with markedly reduced effective work. Early shortening occurs because of the unloading effect of dyskinetic myocardium, which acts as an elastic slack element during isovolumic contraction [15]. Within each cardiac cycle, regional P/L loops move in an opposite direction and asynchronously. Each region therefore produces a different contribution to global ejection.

### 2E. Neurohumoral Activation

Remodeling involves concomitant neurohumoral activation, which is characterized by increased plasma norepinephrine [NE], renin (PRA) [16], angiotensin [A-II] [17] and BNP [18]. The schematic details the genesis of LV dysfunction in a dilated ventricle [19].

## 3. THE IMPACT OF VOLUME

LV dilatation (defined as >20% increase in end diastolic volume) occurs in approximately 20% of infarcted patients as shown by Gaudron [20], Bolognese [21], the TOAT study [22] and the SAVE study [23]. Normal left ventricular endsystolic volume index [LVESVI] is less than 30 ml/m^2^. The hallmark insight into the importance of using ventricular volume rather than EF to prognosticate post MI survival comes from White’s [24] analysis, which showed that patients with LVESVI >60 ml/m^2^ have approximately a fivefold increase in mortality compared with those with normal volumes after infarction.

The GUSTO I trial showed that among infarction patients with successful thrombolysis, 17% had progressive LV enlargement above 40 ml/m^2^. Mortality at one year was 16% among those with LVESVI 40 to 50 ml/m^2^, 21% with LVESVI 50 to 60 ml/m^2^ and 33% when LVESVI >60 ml/m^2^ [25].

## 4. RESPONSE TO CORONARY SURGERY

Ventricular size determines prognosis after successful revascularization. Wall motion, ejection fraction and LVESVI improve if initial LVESVI is <75 ml/m^2^, but progressively deteriorate when pre-operative LVESVI is >75 ml/m^2^ despite open grafts [26].

The importance of volume reduction is emphasized by Yamaguchi *et al.* [27] who analyzed the impact of LV volume on operative results for CABG in ischemic cardiomyopathy, and concluded that LVESVI greater than 100 ml/m^2^ independently predicted postoperative heart failure.

The underlying principle of the STICH (Surgical Treatment for Ischemic Heart failure) trial involves recognition of the potentially lethal complications of enlarging ventricular volume.

## 5. IMPACT OF SHAPE

LV shape change from elliptical to spherical, reduces systolic torsion, as the myofibrils shift from their normal oblique axis toward a more transverse direction. The normal myofibril shortening of 15% generates a global EF of only 30% in spherical ventricles, compared to an EF of 60% in elliptical ventricles with natural torsion [28].

Large anterior infarctions compromise LV torsion, which is the fundamental mechanism “squeezing” the LV cavity to cause ejection. Compromised LV torsion thus leads to heart failure. Posterior MI’s produce posterior papillary muscle dysfunction and/or posterior wall motion abnormalities, thereby causing MR.

## 6. SVR ANATOMY

Torrent-Guasp proposed a challenging and very important anatomic concept in which both ventricles are considered to consist of a single myocardial band extending from the right ventricular muscle just below the pulmonary artery to the left ventricular muscle where it attaches to the aorta [29, 30]. The structural components include a horizontal or transverse fibre orientation for the basal loop that surrounds the right and left ventricles, and a change in fibre direction to form an apical helix with descending and ascending segments. This configuration equalizes stresses and strains across the ventricle [31, 32].

## 7. GEOMETRY and FLOW DYNAMICS

‘’ Blood flow in the heart is spirally twirled ‘’

            Leonardo da Vinci

The deformation responsible for contractile strain increases from the widened base to the helical apex [33]. The pattern of ejection and filling are related to a sequential twisting of the LV to eject and a rapid untwisting to suction venous return and allow rapidly filling [34].

There is strong evidence that the transmission of flow into the aorta is helically shaped [35]. Consequently, spatial motion of flow in the LV and aorta closely resembles the pattern evident in typhoons or tornadoes. In contrast, there is destruction of helical flow in the dilated failing heart.

The elliptical shape formed by the overlapping ascending and descending segments of the apical loop accounts for the natural helix formation. With LV dilatation, the architecture of oblique apical loop fibres becomes more transverse, thereby resembling the horizontal fibre orientation of the basal loop. The value of reconstructing the helix to generate an elliptical shape [30] is emphasized because factors responsible for ejection and suction during the cardiac cycle are linked to this apical loop.

### 7A. Functional Geometry

The cardiac architecture of the healthy heart is Gothic (elliptical), while that of the sick heart is Romanesque (spherical) [36]. Ingels [28] emphasized the importance of the ‘opposing force couples’ from subendocardial fibres disposed in a right handed helix and subepicardial fibres in a left handed helix. The summation of these force couples generates the torsional deformation of the left ventricle about its long axis.

## 8. PATHOLOGICAL SUBSTRATES

LV sphericity is the common architectural event following any disease that causes global stretch and results from one of three conditions.

- SVR in ischemic disease is based on recognizing the borders of the scar. The junction between scar and surrounding muscle determines patch placement. However, this transition zone ignores remote muscle dilatation, resulting in a large residual volume of dilated remote muscle and accounts for the poor long-term prognosis following restoration, if pre-operative end systolic volume index is >100-120 ml/m2 [37]. In ventricles without scar, SVR is not always performed. Isolated CABG, in these circumstances is associated with large post-operative volumes and poor outcomes [26].
- The absence of visible myocyte pathology in dilated hearts with valvular disease has so far precluded routine use of SVR. Outcomes remain poor following replacement or repair when pre-operative EF is <40% [38].
- Non-ischemic cardiomyopathy from direct muscular involvement may stem from inflammation or sarcoidosis. Non-ischemic cardiomyopathy is a heterogenous disease involving predominantly the septum and lateral wall [39]. Procedures such as partial left ventriculectomy failed due to the erroneous assumption that the disease was homogenous, with the consequent resection of healthy contractile lateral wall muscle in several cases.

## 9. THERAPEUTIC ALTERNATIVES

The introduction of angiotensin-converting enzyme inhibitors, beta-blockers, aldosterone antagonists, HMG CoA reductase inhibitors, antiplatelet therapy, cardiac resynchronization and implantable cardioverter defibrillators has reduced mortality from heart failure.

Even with optimal medical management, the mortality in patients with advanced HF is reportedly 40% to 50% at 1 to 2 years. MacIntyre and colleagues demonstrated that mortality among all-comers with CHF at 2 year approaches 45% [40]. In the Randomized Evaluation of Mechanical Assistance for the Treatment of Congestive Heart Failure (REMATCH) trial, patients in NYHA IV demonstrated 25% survival at 1 year and 8% survival at 2 years with optimal medical management.

## 10. SURGICAL ALTERNATIVES

### 10 A. Coronary Revascularization

Patients with ischemic cardiomyopathy and a substantial amount of viable myocardium and a high end-systolic volume due to LV remodeling have a decreased likelihood of improvement of global function following myocardial revascularization or relief of heart failure symptoms.

The five-year survival of patients with EF ≤35% is 50% to 65% [41]. In one study, CABG mortality was 27% if LV end-diastolic diameter was ≥81 mm [42]. CHF symptoms are common after CABG for ischemic cardiomyopathy. Yamaguchi's analysis of CABG for patients with EF ≤30% showed a five-year survival of 54% with preoperative LVESVI ≥100 ml/m^2^ compared to 85% if LVESVI was ≤100 ml/m^2^. CHF at five years was seen in 69% of patients with the larger hearts versus 15% with the smaller ones [43]. Luciani et al reviewed 167 patients who underwent CABG with a mean EF of 28%. At five years, 60% of patients continued to have signs and symptoms of CHF, demonstrating the limitations of CABG alone [44].

### 10 B. Mitral Valve Repair or Replacement

Patients with ischemic cardiomyopathy undergoing mitral procedures have a five-year mortality of approximately 50% [45]. Recurrence of CHF occurs in one-third of patients by five years and is the most common cause of death, presumably related to continuing dysfunction of the unmodified ventricle [38].

### 10 C. Partial Left Ventriculectomy

In 1995 Batista introduced partial left ventriculectomy to treat patients with non-ischemic dilated cardiomyopathy, by using a concept based on Laplace's law but results were variable due to non-specific exclusion of the lateral wall [46], which might have contained important viable muscle. Results were marred by (1) high surgical mortality, (2) concerns of diastolic dysfunction, (3) recurrent heart failure and (4) ventricular arrhythmias. While PLV had been performed to treat dilated cardiomyopathy [DCM], the hospital mortality varied between 1.9 and 27%, with a mean mortality of 17%. The causes of hospital death were CHF, hemorrhage, residual MR and multi-organ failure.

### 10 D. Ventricular Assist 

In the REMATCH trial, less than 10% of patients survived to three years in the LVAD group, compared with no survivors among patients treated medically [47]. The five-year survival after cardiac transplantation is 70%.

### 10 E. Cardiac Transplantation

Cardiac transplantation offers the greatest benefit for patients with refractory heart failure, with a 1-year survival rate of 86% and a 5-year survival rate of 69%. With a limited donor organ pool and an increasing incidence of heart failure, this approach has limited application.

## 11. SURGICAL VENTRICULR RESTORATION

In ischemic cardiomyopathy, the spherical geometry of the globally hypokinetic dilated heart leads to malfunction; hence contractility is not improved by simply restoring blood supply. Failure to change the natural course of progression of heart failure by isolated correction of coronary and/or valve pathology suggests that a ‘valve and ventricle’ approach should be considered. Neurohumoral factors are not deranged without a triggering event; the elimination of the trigger (e.g., surgical reduction of pathologic cardiac wall stress) may result in neurohumoral inhibition.

In 1985, Vincent Dor described an original surgical technique, the Endoventricular Circular Patch Plasty [48] and subsequently reported excellent clinical and hemodynamic results of this procedure [49].

Surgical ventricular restoration (SVR) is a surgical option designed to reverse the maladaptive morphologic changes of postinfarction ventricular remodeling, by restoring LV volume and a more normal elliptical shape to the LV, thereby reducing myocardial wall stress and improving ventricular function. SVR includes complete revascularization, LV reconstruction to restore near-normal shape and volume and when necessary, mitral valve repair, in addition to surgery for ventricular tachycardia [VT]. This approach to HF has been considered the ‘triple V’ since the operation corrects the abnormal vascular, valvular and ventricular components of the heart failure process.

## 12. SVR EVOLUTION

In the recent update of the European Society of Cardiology Guidelines for the diagnosis and treatment of chronic heart failure, LV aneurysmectomy is indicated in patients with large, discrete LV aneurysms who develop heart failure.

SVR as described by Dor and associates, was developed as a more physiologic repair of LV aneurysm, compared with simple linear repair, but was later applied to ischemic cardiomyopathy without the classic signs of the true aneurysm.

Dor demonstrated that equivalent results could be achieved with SVR even in patients without discrete ventricular aneurysms.

Dor [50] recognized that the adverse effects of remodeling on the remote non-infarcted myocardium were similar for akinesia and dyskinesia and was the first to utilize the endocardial patch plasty procedure for both morphologies.

## 13. SVR INDICATIONS

Indications for SVR include:

1. Refractory heart failure in NYHA class III or IV
2. A left ventricle diastolic dimension > 75 mm.
3. LVESVI>60ml/m^2^
4. LVEDVI>100ml/m^2^
5. Previous anterior MI.
6. LV dysfunction with regional asynergy (either akinetic or dyskinetic) greater than 35% of the ventricular perimeter.
7. Ventricular arrhythmias and/or angina.
8. For patients who are asymptomatic despite post-infarction LV dysfunction, serial echocardiographic studies could be performed to detect the first signs of deterioration (i.e., progressive LV enlargement or decline in EF).

## 14. CONTRAINDICATIONS

The following are contraindications:

- Severe right ventricular dysfunction (absolute)
- Severe pulmonary hypertension not associated with MR (relative).
- Severe regional asynergy without LV dilatation (absolute).
- Restrictive diastolic pattern associated with high functional class and MR (absolute).

An entirely akinetic LV with no increase in systolic wall thickness

Reduced EF is not a contraindication, but an increased use of IABP should be anticipated. With severe and diffuse cardiac dysfunction, a stress dobutamine stress echo could be performed. SVR is offered if contractility improves.

## 15. EXTENDED INDICATIONS

Studies have shown that SVR can benefit patients with severe left ventricular dysfunction (EF <20%), multiterritory MI, and pulmonary hypertension [PHTN], which were previously considered to be contraindications to SVR [51 - 53]. The survival of NYHA IV patients undergoing SVR is lower than those with milder HF, but still exceeds that of medically managed patients [40, 54].

SVR has also been offered to patients with multiterritory MI. A critical amount of viable myocardium is considered necessary for a successful outcome regardless of the location of infarction. For instance, patients with < 50% involvement of the lateral wall showed similar improvements in cardiac function and survival when compared with those without lateral wall MI’s. When lateral wall involvement exceeded 50%, there were differences in clinical outcomes [55].

PHTN has been shown to be a predictor of death in patients with ischemic cardiomyopathy. However, improved LV function after SVR reduces the load on the pulmonary vasculature, and lowers pulmonary resistance and pressures.

A consideration of the real world application of ventricular restoration procedures reveals that the overall results do not match those seen in large volume centres of expertise, and consequently the premature expansion of such indications outwith such centres of expertise could be potentially detrimental and may not yield equivalent outcomes in borderline patients.

## Figures and Tables

**Fig. (1):**
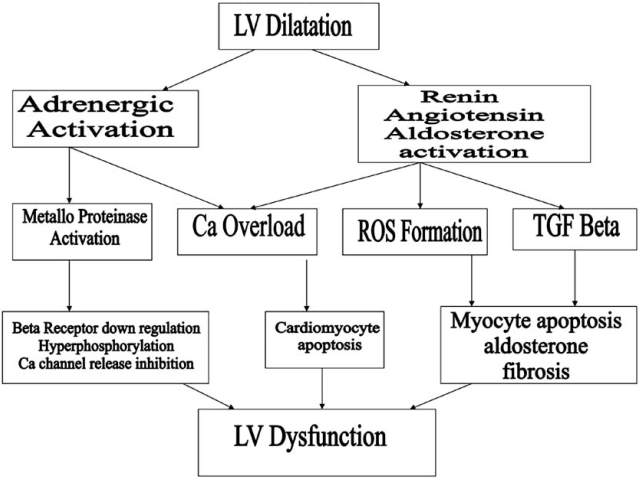
Neurohumoral model.

## ABBREVIATIONS

AII: = Angiotensin II
BNP: = Brain natriuretic peptide
CABG: = Coronary artery bypass grafting
CAD: = Coronary artery disease
CHF: = Congestive heart failure
HF: = Heart failure
ICMPY: = Ischemic cardiomyopathy
IHD: = Ischemic heart disease
EF: = Ejection fraction
EVCPP: = Endoventricular circular patch plasty
DCM: = Dilated cardiomyopathy
GUSTO Trial: = Global Utilization of Streptokinase and Tissue Plasminogen Activator for Occluded Coronary Arteries
IABP: = Intra aortic balloon pump
ICD: = Implantable cardioverter defibrillator
LAD: = Left anterior descending artery
LCX: = Left circumflex artery
LV: = Left Ventricle
LMI: = Lateral wall myocardial infarction
LVA: = Left ventricular aneurysm
LVESVI: = Left ventricular end systolic volume index
LVESV: = Left ventricular end systolic volume
MI: = Myocardial infarction
MR: = Mitral regurgitation
MRI: = Magnetic resonance imaging
NE: = Norepinephrine
NT-pro-BNP: = N-terminal portion of proBNP
NYHA: = New york heart association
PHTN: = Pulmonary hypertension
PLV: = Partial left ventriculectomy
PRA: = Plasma renin assay
PTCA: = Percutaneous transluminal coronary angioplasty
REMATCH: = Randomized evaluation of mechanical assistance for the treatment of congestive heart failure
RESTORE Trial: = Reconstructive Endoventricular Surgery returning Torsion Original Radius Elliptical shape to the left ventricle
RCA: = Right coronary artery
RV: = Right Ventricle
SAVE procedure: = Septal Anterior Ventricular Exclusion
SAVE trial: = Survival and ventricular enlargement
STICH: = Surgical treatment for ischemic heart failure
SVR: = Surgical ventricular restoration
VT: = Ventricular tachycardia
TOAT: = Open artery trial
